# The effects of race/ethnicity and physician recommendation for physical activity on physical activity levels and arthritis symptoms among adults with arthritis

**DOI:** 10.1186/s12889-021-11570-6

**Published:** 2021-08-18

**Authors:** Jeremy Huckleby, Faustine Williams, Rose Ramos, Anna María Nápoles

**Affiliations:** 1 Medical Resident Scholar Program, National Institutes of Health and National Institute on Minority Health and Health Disparities, National Institutes of Health, 9000 Rockville Pike, Building 3, Floor 5, Room E08, Bethesda, MD 20892 USA; 2 Division of Intramural Research, National Institute on Minority Health and Health Disparities, National Institutes of Health, 9000 Rockville Pike, Building 3, Floor 5, Room E08, Bethesda, MD 20892 USA

**Keywords:** Arthritis, Physical activity, Health disparities, Physician advice, Race/ethnicity

## Abstract

**Background:**

Among U.S. adults with physician-diagnosed arthritis, we examined the association of 1) participant race/ethnicity with meeting physical activity guidelines and arthritis symptoms, and 2) the association of receipt of a physician exercise recommendation with physical activity levels and arthritis symptoms, and whether race/ethnicity moderates these associations.

**Methods:**

Retrospective, cross-sectional study of National Health Interview Survey pooled data from 2002, 2006, 2009, and 2014 from 27,887 U.S. adults aged ≥18 years with arthritis. Outcomes were meeting aerobic (yes/no) and strengthening guidelines (yes/no), arthritis-associated activity limitations (yes/no) and arthritis-related pain (0–10; higher score = more pain). Predictors were race/ethnicity (White, African American, Latino, and Asian) and receipt of physician recommendation for exercise (yes/no). Covariates included demographic and health characteristics.

**Results:**

Adjusting for covariates, African Americans were more likely (AOR = 1.27; 95% CI 1.12, 1.43) and Asians were less likely (AOR = 0.75; 95% CI 0.61, 0.92) than Whites to meet muscle strengthening activity guidelines. Compared to Whites, African Americans (B = 0.48; 95% CI 0.24, 0.72) and Latinos (B = 0.44; 95% CI 0.15, 0.72) reported more severe, while Asians reported less severe (B = -0.68; 95% CI -1.22, − 0.14) joint pain. Controlling for covariates, physician exercise recommendation was associated with meeting aerobic (AOR = 1.20; 95% CI 1.11, 1.30) and strengthening (AOR = 1.21; 95% CI 1.11, 1.33) guidelines, regardless of race/ethnicity except for a weak negative association with meeting strengthening guidelines (AOR = 0.85; CI 0.74–0.99) among Latinos.

**Conclusions:**

Disparities in pain exist for African Americans and Latinos with arthritis. Physician exercise recommendation is critical among patients with arthritis to relieve symptom burden.

## Background

In 2015, arthritis affected 23% (54.4 million) of U.S. adults, [1] and is estimated to affect 78.4 million by 2040 [2]. The prevalence of disability due to arthritis is expected to increase from 22.7 to 34.6 million Americans by 2040, a 52% increase from 2012 [2]. Between 2013 and 2015, the average prevalence of arthritis was similar between Whites and African Americans (22.6% vs 22.2%) but lower among Latinos (15.7%) and Asians (11.8%) [1]. However, arthritis-related activity limitations and pain were significantly higher among African Americans, Latinos, mixed-race, and American Indian/Native Alaskan populations compared to Whites [1]. Effective interventions to address racial/ethnic disparities in symptom burden are warranted.

Physical activity (PA) is a safe, non-pharmacologic, evidence-based intervention to reduce arthritis symptoms. PA is effective at reducing pain and improving function among persons with moderate-to-severe arthritis and compared to the short-term benefits of nonsteroidal anti-inflammatory drugs and opioids, regular exercise provides more durable benefits [3–8]. Also, exercise improves mood and quality of life in those with chronic pain due to arthritis [9, 10]. Although some evidence suggests that strengthening exercise has a larger effect on pain reduction, [11] both aerobic and strengthening exercises are efficacious in reducing pain and improving function [7]; offering patients a choice between the two may improve adherence [7]. Musculoskeletal organizations such as the American Academy of Orthopedic Surgeons (AAOS) and the Osteoarthritis Research Society International (OASRI) have issued consistent recommendations for regular PA for persons with arthritis [12].

Despite strong evidence that PA is beneficial for people with arthritis, almost one-third of persons with arthritis are completely inactive, and only one-quarter adhere to national PA recommendations [13]. The picture is even bleaker for minorities. African Americans and Latino adults with arthritis were less likely than Whites to meet aerobic physical activity guidelines [14]. In one study among persons with or at risk of knee osteoarthritis, African Americans were 72–76% less likely than Whites to meet PA guidelines [15].

Receiving advice from a physician to exercise is associated with a higher likelihood of meeting aerobic PA guidelines among those with arthritis [16]. In 2011, only 60% of persons with arthritis received a physician recommendation to exercise for relief of arthritis symptoms [16]. Several studies have demonstrated that African Americans and Latinos with arthritis were less likely to receive PA advice from physicians than their White counterparts [12, 17]. It is plausible that physician implicit bias could affect whether a physician gives an exercise recommendation for patients from certain racial/ethnic groups because evidence shows African Americans are less likely to receive appropriate pain management compared to White Americans [18]. Also, patients most likely to benefit (obese/overweight and those with higher pain levels, comorbidities, and activity limitations) were less likely to have received a physician recommendation to exercise [19]. Yet, studies support recommending PA to reduce arthritis symptoms regardless of patient profile, including radiologic severity and pain levels [19]. Disparities in receipt of physician recommendation for PA for arthritis symptoms could help explain disparities in symptom burden.

Studies examining the role of physician recommendation for PA among persons with arthritis have been limited in that they only asked about PA in general, rather than differentiating between aerobic and muscle strengthening activities. It is important for physicians to know if there is systematic variation in patient preferences in type of PA, as this information could be used to increase adherence [7].

Using data from the National Health Interview Survey (NHIS), the objectives of this study were to examine: 1) the association of participant race/ethnicity with meeting physical activity guidelines (aerobic and strengthening) and arthritis symptoms (arthritis-associated activity limitations and severity of joint pain); and 2) the association of receipt of a physician recommendation to exercise with meeting PA guidelines (aerobic and strengthening) and arthritis symptoms (arthritis-associated activity limitations and arthritis-related pain), and whether race/ethnicity moderates these associations.

## Methods

### Study design and sample

This study included 27,877 adults; 64.3% were women and the mean age was 60.9 years (SD = 15.1) (Table 1). NHIS data was pooled from 2002, 2006, 2009, and 2014, linking the Adult Core sample file and Person file for each year. The NHIS is an ongoing, multistage probability cross-sectional in-person household survey of a nationally representative sample of the U.S. noninstitutionalized population residing in all 50 U.S. states and the District of Columbia [20]. We selected adults (ages 18 years and older) with self-reported arthritis defined by a “yes” response to the item, “Have you EVER been told by a doctor or other health professional that you have some form of arthritis, rheumatoid arthritis, gout, lupus, or fibromyalgia?” Responses of “no” and those with unknown arthritis status were excluded. For the remainder of the manuscript, the phrase “doctor or other health professional” is referred to as physician. The authors utilized publicly available de-identified data; therefore, this study does not constitute human subjects research.

**Table 1:** Characteristics of U.S. Adults Aged ≥ 18 years with Arthritis by Race/Ethnicity, National Health Interview Survey, 2002, 2006, 2009, and 2014, *N* = 27,887

Characteristics	Total	White	African American	Latino	Asian	
	N (%)	N (%)	N (%)	N (%)	N (%)	*p*-value
*Demographic characteristics*						
Age (in years)						
18–44	4138 (14.8)	2793 (13.8)	674 (15.7)	581 (20.7)	90 (14.6)	**<.0001**
45–64	11,652 (41.8)	8288 (41.1)	1941 (45.3)	1209 (43.1)	214 (34.6)	
≥ 65	12,097 (43.4)	9100 (45.1)	1669 (39.0)	1014 (36.2)	314 (50.8)	
Sex						
Female	17,922 (64.3)	12,646 (62.7)	2990 (69.8)	1906 (68.0)	380 (61.5)	**<.0001**
Male	9965 (35.7)	7535 (37.3)	1294 (30.2)	898 (32.0)	238 (38.5)	
Education						
< high school	5769 (20.8)	3061 (15.2)	1294 (30.5)	1276 (46.0)	138 (22.5)	**<.0001**
high school graduate	8236 (29.7)	6215 (30.9)	1242 (29.3)	642 (23.1)	137 (22.4)	
Technical or some college	8018 (28.9)	6123 (30.5)	1169 (27.5)	592 (21.3)	134 (21.9)	
> College degree	5692 (20.5)	4685 (23.3)	539 (12.7)	265 (9.5)	203 (33.2)	
*Health characteristics*						
Smoking Status						
Never	13,449 (48.6)	9197 (45.9)	2205 (52.0)	1634 (58.6)	413 (67.2)	**<.0001**
Former	8889 (32.1)	6941 (34.6)	1083 (25.5)	719 (25.8)	146 (23.7)	
Current	5355 (19.3)	3911 (19.5)	954 (22.5)	434 (15.6)	56 (9.1)	
Body mass index						
< 18.5, underweight	395 (1.5)	305 (1.6)	47 (1.1)	21 (0.8)	22 (3.6)	**<.0001**
18.5–24.99, normal weight	7248 (27.1)	5631 (29.1)	795 (19.4)	527 (19.7)	295 (48.8)	
25–29.9, overweight	9067 (34.0)	6667 (34.5)	1222 (29.8)	974 (36.4)	204 (33.8)	
≥ 30, obese	9989 (37.4)	6716 (34.8)	2036 (49.7)	1154 (43.1)	83 (13.7)	
Self-rated health						
poor/fair	8555 (30.7)	5290 (26.2)	1891 (44.2)	1200 (42.8)	174 (28.2)	**<.0001**
good/very good/excellent	19,308 (69.3)	14,873 (73.8)	2390 (55.8)	1602 (57.2)	443 (71.8)	
Number of co-morbidities^1^						
0	6686 (24.6)	5002 (25.4)	756 (18.1)	758 (27.9)	170 (28.8)	**<.0001**
1–2	15,104 (55.5)	10,843 (55.0)	2456 (58.9)	1469 (54.0)	336 (56.9)	
≥ 3	5419 (19.9)	3883 (19.7)	957 (23.0)	494 (18.2)	85 (14.4)	
Psychological distress^2^						
none to mild (0–4)	19,407 (71.3)	14,381 (72.9)	2868 (68.6)	1714 (62.9)	444 (74.9)	**<.0001**
moderate (5–12)	6037 (22.2)	4182 (21.2)	1000 (23.9)	734 (26.9)	121 (20.4)	
severe (≥ 13)	1774 (6.5)	1156 (5.9)	312 (7.5)	278 (10.2)	28 (4.7)	
*Outcomes*						
Received clinician recommendation to exercise to help arthritis						
Yes	15,799 (56.9)	11,068 (55.1)	2645 (62.0)	1713 (61.3)	373 (60.4)	**<.0001**
No	11,982 (43.1)	9031 (44.9)	1623 (38.0)	1083 (38.7)	245 (39.6)	
Severity of point Pain						
Mild (0–3)	11,503 (41.7)	8785 (44.0)	1400 (33.3)	1013 (36.6)	305 (50.3)	**<.0001**
Moderate (4–7)	10,817 (39.2)	8107 (40.6)	1494 (35.5)	991 (35.8)	225 (37.1)	
Severe (8–10)	5243 (19.0)	3086 (15.4)	1314 (31.2)	767 (27.7)	76 (12.5)	
Arthritis-associated activity limitations						
Yes	11,487 (41.3)	7893 (39.2)	2051 (48.0)	1312 (46.9)	231 (37.5)	**<.0001**
No	16,354 (58.7)	12,257 (60.8)	2225 (52.0)	1487 (53.1)	385 (62.5)	
Met guideline for aerobic physical activity^3^						
Yes	9151 (33.9)	7124 (36.5)	1092 (26.2)	716 (26.1)	219 (36.0)	**<.0001**
No	17,879 (66.1)	12,387 (63.5)	3076 (73.8)	2027 (73.9)	389 (64.0)	
Met guideline for strengthening physical activity ^4^						
Yes	4500 (16.3)	3507 (17.5)	572 (13.5)	313 (11.2)	108 (17.6)	**<.0001**
No	23,118 (83.7)	16,478 (82.5)	3666 (86.5)	2470 (88.8)	504 (82.4)	

^1^ Count of the following conditions: asthma, cancer, chronic obstructive pulmonary disease, heart disease, hepatitis, diabetes, kidney disease, hypertension, psychological distress, and stroke

^2^ Psychological distress was assessed using the Kessler-6 and recommended cutpointss [21]

^3^ Using 2018 Physical Activity Guidelines for Americans recommendation of 150 min per week of moderate aerobic physical activity, 75 min per week of vigorous aerobic physical activity or an equivalent combination of moderate and vigorous aerobic physical activity [22]

^4^ Using 2018 Physical Activity Guidelines for Americans recommendation of two or more days per week of calisthenics, resistance training, weightlifting or any activities that require muscle tension against a weight or force (i.e., strengthening physical activity) [22]

### Measures

#### Outcomes

##### Meets aerobic physical activity guidelines

The Health and Human Services (HHS) 2018 Physical Activity Guidelines for Americans 2nd edition recommend 150 min/week of moderate aerobic PA (e.g., brisk walking) or 75 min/week of vigorous aerobic PA (e.g., running) or an equivalent combination for all adults [22]. Individuals reported weekly frequency and duration of moderate and vigorous aerobic PA. Total aerobic PA was assessed by combining moderate and vigorous aerobic PA, where 1 min of vigorous aerobic PA is equivalent to 2 min of moderate aerobic PA [14]. Those reporting ≥150 min/week of total aerobic PA were considered to have met aerobic PA guidelines.

##### Meets muscle strengthening physical activity guidelines

The HHS 2018 guidelines for muscle strengthening PA recommend strengthening activities for ≥2 days per week. For our analysis, individuals that report lifting weights or doing calisthenics ≥2 times/week were considered to meet muscle strengthening PA guidelines.

Both aerobic and strengthening PA guidelines are the same for older adults (65 years or older). However, due to the higher burden of chronic diseases, and increased risk of fall injuries in this group, for those aged > 65 years, it is added that training should include balance exercises, and physical activity intensity and total time should be limited to what their abilities and conditions allow [22].

##### Arthritis-associated activity limitation

This variable was assessed using the item “Are you now limited in any of your usual activities because of your arthritis or joint symptoms?*”* Response options were yes/no.

##### Severity of arthritis-related pain

Individuals were asked if they experienced symptoms of pain, aching, or stiffness in or around a joint in the past 30 days. Those responding “yes” were asked to rate their average joint pain during the past 30 days on a scale of 0 to 10; 0 = no pain or aching and 10 = pain and aching as bad as it can be. Those responding “no” to the initial question were considered to have no pain and assigned a value of 0 (no pain); thus scores ranged from 0 to 10 with a higher score indicating more pain.

#### Predictor variables

##### Self-reported race/ethnicity

The race/ethnicity item used response options of: Hispanic, non-Hispanic White, Non-Hispanic Black, Non-Hispanic Asian, and Non-Hispanic/all other races. Hereafter, we refer to these groups as Latino, White, African American, and Asian. Respondents reporting non-Hispanic/all other races were dropped from the analyses due to insufficient sample size for an adequately powered analysis.

##### Receipt of physician recommendation to exercise to help arthritis

This variable (yes/no) was assessed using the question, *“*Has a doctor or other health professional EVER suggested physical activity to help your arthritis or joint symptoms?” Those with missing responses were excluded.

#### Covariates

Demographic covariates included age (18–44, 45–64, 65, or > 65 years), sex (male or female), educational attainment (less than high-school degree, high-school graduate, technical college/some university, four year college degree or higher), marital status (yes/no), employment status (yes/no), annual household income (0-$34,999, $35,000-64,999, and ≥ $65,000), health insurance (yes/no), having a usual source of care (yes/no), and U.S. region (Northeast, Midwest, South, or West).

Health covariates included: smoking status (never, former, or current); body mass index (BMI calculated from self-reported weight and height as a continuous measure (weight in kg/height in m^2^) categorized as underweight (< 18.5), normal weight (18.5–24.9), overweight (25.0–29.9), or obese (> 30); self-rated health (excellent/very good/good versus fair/poor); and number of comorbidities (0, 1–2, or ≥ 3) as a count of the following conditions: asthma, cancer, chronic obstructive pulmonary disease, heart disease, hepatitis, diabetes, kidney disease, hypertension, psychological distress, and stroke; and psychological distress assessed with the Kessler-6 and categorized into 3 levels based on the sum score: none/mild (0–4), moderate (5–12), and severe (≥13) [21, 23].

### Statistical analysis

Descriptive statistics were used to examine the distributions of demographic, health characteristics, and outcomes by race/ethnicity**.** Chi-square tests were used to assess bivariate differences in demographic factors, health characteristics, and outcomes by race/ethnicity.

Multivariable logistic regression was used to analyze the association of race/ethnicity and receipt of physician recommendation on the odds of meeting aerobic and strengthening PA guidelines, and arthritis activity limitation). Additionally, we assessed whether the association of receiving physician recommendation to exercise with the odds of meeting aerobic and strengthening PA guidelines and reporting arthritis activity limitations were moderated by race/ethnicity (included an interaction term for race/ethnicity x receipt of physician recommendation in the model). Covariates included demographic characteristics (age, sex, education, marital status, income, employment, health insurance, having a usual source of care, and region) and health characteristics (smoking status, BMI, self-rated health, number of comorbidities, and psychological distress).

Multivariable linear regression was used to analyze the association of race/ethnicity and receipt of physician recommendation on joint pain severity, and whether the association physician recommendation with pain severity was moderated by race/ethnicity **(**interaction term for race/ethnicity x receipt of physician recommendation was included in the model). Covariates included demographic characteristics (age, sex, education, marital status, income, employment, health insurance, having a usual source of care, and region) and health characteristics (smoking status, BMI, self-reported health, comorbidities, psychological distress).

With approximately 13–15% missing response rates on our outcome variables we used weighted hotdeck imputation to account for these missing responses while also accounting for study weights. Using weighted hotdeck imputation with 20 donors, SAS proc. MIAnalyze was used to account for the between-imputation variance.

The NHIS sampling weights for the Sample Adult Core were used for all analyses to generate nationally representative population estimates. SAS/STAT software PROC SURVEY SELECT was utilized to insure correct estimates and standard errors.

## Results

### Descriptive analysis

African Americans had higher rates of current smoking, obesity, poor/fair self-reported health, comorbidities and severe psychological distress than all other groups (Table 1). Latinos had higher rates of obesity, poor/fair self-reported health status and severe psychological distress compared to Whites and Asians (Table 1).

Rates of receipt of physician recommendation were highest among African Americans (62%) and Latinos (61.3%), followed by Asians (60.4%) and Whites (55.1%). Almost 60% of the sample reported moderate to severe joint pain and 41.3% reported arthritis-associated activity limitations. African Americans and Latinos were more likely to report moderate to severe joint pain and arthritis-associated activity limitations than Whites. Overall, 34% met aerobic and 16% met muscle strengthening PA guidelines, with rates being equal among Whites and Asians but lower among African Americans and Latinos (Table 1).

### Multivariable analyses

#### Meeting physical activity guidelines

Adjusting for demographic and health-related factors, there were no differences in the odds of meeting aerobic PA guidelines by race/ethnicity (Table 2). Controlling for demographic and health-related factors, compared to Whites, African Americans were more likely to meet strengthening guidelines (AOR = 1.27; 95% CI 1.12, 1.43) while Asians were less likely than Whites to meet muscle strengthening (AOR = 0.75; 95% CI 0.61, 0.92) guidelines.

**Table 2:** Association of Race/Ethnicity and Receipt of Physician Recommendation on Meeting Aerobic and Strengthening Physical Activity Guidelines, and Arthritis-Associated Activity Limitations among U.S. Adults Aged ≥ 18 years with Arthritis, National Health Interview Survey, 2002, 2006, 2009, and 2014

	Meets Aerobic Physical Activity Guidelines^1^		Meets Muscle Strengthening Physical Activity Guidelines^1^		Has Arthritis-Associated Activity Limitations^1^	
	Unadjusted OR (95% CI)	Adjusted OR (95% CI)	Unadjusted OR (95% CI)	Adjusted OR (95% CI)	Unadjusted OR (95% CI)	Adjusted OR (95% CI)
		*N* = 23,722		*N* = 24,208		*N* = 24,253
Race/ethnicity (ref: White)						
African American	**0.82 (0.74–0.90)**	1.08 (0.96–1.21)	0.94 (0.84–1.05)	**1.27 (1.12–1.43)**	**1.24 (1.15–1.35)**	1.03 (0.93–1.14)
Latino	**0.83 (0.75–0.93)**	0.94 (0.84–1.06)	**0.75 (0.66–0.85)**	0.95 (0.83–1.10)	**1.14 (1.03–1.25)**	0.95 (0.84–1.08)
Asian	1.13 (0.91–1.39)	0.80 (0.64–1.00)	1.17 (0.98–1.41)	**0.75 (0.61–0.92)**	0.84 (0.70–1.00)	1.01 (0.79–1.28)
Received Physician Recommendation for Exercise to Help Arthritis (ref: No)						
Yes	**1.07 (1.04–1.11)**	**1.20 (1.11–1.30)**	**1.13 (1.09–1.18)**	**1.21 (1.11–1.33)**	**1.30 (1.26–1.34)**	**1.27 (1.17–1.39)**
Interaction term for Race (ref: White) x Received Physician Recommendation						
African American x Received Physician Recommendation		0.94 (0.85–1.04)		0.97 (0.85–1.11)		0.96 (0.86–1.06)
Latino x Received Physician Recommendation		1.05 (0.94–1.17)		**0.85 (0.74–0.99)**		1.03 (0.91–1.16)
Asian x Received Physician Recommendation		1.09 (0.90–1.32)		1.23 (0.98–1.54)		1.01 (0.80–1.27)
*Health Characteristics*						
Smoking Status (ref: Never)						
Former	**1.12 (1.07–1.18)**	**1.14 (1.08–1.20)**	**1.17 (1.10–1.25)**	**1.20 (1.12–1.29)**	**0.94 (0.90–0.98)**	0.97 (0.92–1.02)
Current	**0.81 (0.76–0.86)**	**0.84 (0.78–0.90)**	**0.70 (0.65–0.76)**	**0.73 (0.66–0.79)**	**1.20 (1.14–1.27)**	1.04 (0.98–1.11)
BMI (ref: < 25, underweight/normal)						
25–29.9, overweight	**1.12 (1.07–1.17)**	**1.08 (1.03–1.14)**	1.02 (0.96–1.09)	0.99 (0.93–1.06)	**0.90 (0.86–0.93)**	0.95 (0.91–1.00)
≥ 30, obese	**0.76 (0.72–0.79)**	**0.77 (0.73–0.81)**	**0.72 (0.68–0.76)**	**0.72 (0.68–0.77)**	**1.34 (1.28–1.39)**	**1.19 (1.14–1.26)**
Self-reported health status (ref: good, very good, excellent)						
poor/fair	**0.54 (0.52–0.56)**	**0.67 (0.64–0.70)**	**0.58 (0.55–0.62)**	**0.74 (0.70–0.79)**	**2.26 (2.18–2.35)**	**1.74 (1.67–1.81)**
Number of Comorbidities (Ref: 0)						
1–2	**1.06 (1.01–1.10)**	1.02 (0.97–1.08)	1.01 (0.96–1.07)	0.99 (0.93–1.05)	**0.90 (0.87–0.94)**	0.96 (0.92–1.00)
≥ 3	**0.60 (0.57–0.64)**	**0.87 (0.82–0.93)**	**0.64 (0.59–0.69)**	**0.89 (0.82–0.96)**	**1.96 (1.85–2.07)**	**1.25 (1.18–1.33)**
Psychological Distress (Ref: none to mild (0–4))						
moderate (5–12)	1.08 (1.00–1.16)	1.07 (0.99–1.16)	1.07 (0.97–1.17)	1.05 (0.95–1.16)	1.03 (0.97–1.10)	1.05 (0.98–1.12)
severe (≥ 13)	**0.56 (0.50–0.63)**	**0.76 (0.67–0.85)**	**0.62 (0.53–0.71)**	0.86 (0.73–1.01)	**2.48 (2.25–2.73)**	**1.64 (1.47–1.83)**

^**1**^ Controlling for demographic factors (age, sex, education, marital status, income, employment, health insurance, having a usual source of care, and U.S. region) and health characteristics (smoking status, body mass index, self-rated health, number of comorbidities, and psychological distress)

Adjusting for demographic and health covariates, receipt of physician recommendation to exercise was associated independently with meeting aerobic (AOR = 1.20; 95% CI 1.11, 1.30) and muscle strengthening (AOR = 1.21; 95% CI 1.11,1.33) guidelines (Table 2). There was a positive association between physician recommendation to exercise and meeting aerobic and muscle strengthening guidelines regardless of race/ethnicity (i.e., interaction terms for race/ethnicity x physician recommendation were not significant), except that there was a weak and negative interaction between Latino ethnicity and physician recommendation for the outcome of meeting strengthening guidelines.

Health characteristics associated with lower odds of meeting aerobic PA guidelines were current smoking status (versus never; AOR = 0.84; 95% CI 0.78, 0.90), obesity (versus BMI < 25; AOR = 0.77; 95% CI 0.73, 0.81), poor/fair self-rated health (versus good/very good/excellent; AOR = 0.67; 95% CI 0.64, 0.70), > 3 comorbidities (versus none; AOR = 0.87; 95% CI 0.82, 0.93), and severe psychological distress (versus none-mild; AOR = 0.76; 95% CI 0.67, 0.85). Similarly, current smoking (AOR = 0.73; 95% CI 0.66, 0.79), obesity (AOR = 0.72; 95% CI 0.68, 0.77), poor/fair self-reported health (AOR = 0.74; 95% CI 0.70, 0.79), and > 3 comorbidities (AOR = 0.89; 95% CI 0.82, 0.96) were associated with decreased odds of meeting strengthening guidelines, controlling for covariates.

#### Arthritis symptom severity

Although African Americans (OR = 1.24; 95% CI 1.15, 1.35), and Latinos (OR = 1.14; 95% CI 1.03, 1.25) were more likely than Whites to report arthritis-associated activity limitations in unadjusted analyses, racial/ethnic differences were attenuated when controlling for demographic and health-related factors (Table 2).

Receipt of physician recommendation was associated with higher odds of reporting activity limitations (versus no recommendation; AOR = 1.27; 95% CI 1.17, 1.39), controlling for covariates. There were no racial/ethnic differences in the effects of physician exercise recommendation on the odds of reporting arthritis-associated activity limitations (i.e., interaction terms for race/ethnicity x physician recommendation were not significant).

Controlling for covariates, health characteristics that were associated with greater odds of reporting arthritis-associated activity limitations were obesity (AOR = 1.19; 95% CI 1.14, 1.26), poor/fair self-reported health (AOR = 1.74; 95% CI 1.67, 1,81), > 3 comorbidities (AOR = 1.25; 95% CI 1.18, 1.33), and severe psychological distress (AOR = 1.64; 95% CI 1.47, 1.83).

Controlling for covariates, compared to Whites, African Americans (B = 0.48; 95% CI 0.24, 0.72) and Latinos (B = 0.44; 95% CI 0.15, 0.72) reported more severe joint pain (Table 3), while Asians reported less severe pain (B = -0.68; 95% CI -1.22, − 0.14). Controlling for covariates, receipt of a physician recommendation was positively associated with severity of joint pain (B = 0.65; 95% CI 0.55, 0.75) and this relationship did not differ by race/ethnicity (i.e. interaction term for race/ethnicity x physician recommendation was not significant).

**Table 3:** Association of Race/Ethnicity and Receipt of Physician Recommendation on Severity of Joint Pain among U.S. Adults Aged ≥ 18 years with Arthritis, National Health Interview Survey, 2002, 2006, 2009, and 2014

	Severity of Joint Pain^**1**^	
	Unadjusted Beta Estimate (95% CI)	Adjusted Beta Estimate (95% CI)
		*N* = 24,107
Race/ethnicity (ref: Non-Hispanic White)		
African American	**1.06 (0.92, 1.21)**	**0.48 (0.24, 0.72)**
Latino	**0.79 (0.61, 0.97)**	**0.44 (0.15, 0.72)**
Asian	**−0.65 (−0.97, −0.32)**	**−0.68 (−1.22, −0.14)**
Received Physician Recommendation to Exercise to Help Arthritis (ref: No)		
Yes	**0.85 (0.75, 0.95)**	**0.65 (0.55, 0.75)**
Interaction term for Race (ref: White) x Received Physician Recommendation		
African American x Received Physician Recommendation		−0.04 (−0.32, 0.25)
Latino x Received Physician Recommendation		−0.22 (− 0.56, 0.13)
Asian x Received Physician Recommendation		0.39 (−0.24, 1.02)
*Health Characteristics*		
Smoking Status (Ref: Never)		
Former	0.10 (−0.01, 0.20)	0.07 (−0.04, 0.17)
Current	**0.71 (0.57, 0.84)**	**0.29 (0.15, 0.42)**
BMI (Ref: < 25, underweight/normal)		
25–29.9, overweight	**0.23 (0.11, 0.36)**	**0.19 (0.07, 0.31)**
≥ 30, obese	**0.98 (0.84, 1.11)**	**0.54 (0.41, 0.66)**
Self-reported health status (Ref: good, very good, excellent)		
Poor/fair	**2.07 (1.96, 2.17)**	**1.14 (1.01, 1.27)**
Number of comorbidities (Ref: 0)		
1–2	**0.69 (0.57, 0.80)**	**0.29 (0.18, 0.41)**
≥ 3	**1.74 (1.59, 1.88)**	**0.66 (0.51, 0.81)**
Psychological Distress (Ref: none to mild (0–4)		
Moderate (5–12)	**1.52 (1.40, 1.64)**	**0.91 (0.78, 1.03)**
Severe (≥ 13)	**2.80 (2.61, 2.99)**	**1.61 (1.41, 1.80)**

^**1**^ Controlling for demographic factors (age, sex, education, marital status, income, employment, health insurance, having a usual source of care, and U.S. region) and health characteristics (smoking status, body mass index, self-rated health, number of comorbidities, and psychological distress)

Health characteristics that were associated positively with joint pain severity were current smoking (B = 0.29; 95% CI 0.15, 0.42), being overweight (B = 0.19; 95% CI 0.07, 0.31) or obese (B = 0.54; 95% CI 0.41, 0.66), poor/fair self-reported health (B = 1.14; 95% CI 1.01, 1.27), 1–2 comorbidities (B = 0.29; 95% CI 0.18, 0.41) or > 3 comorbidities (B = 0.66; 95% CI 0.51, 0.81), and moderate (B = 0.91; 95% CI 0.78, 1.03) or severe psychological distress (B = 1.61; 95% CI 1.41, 1.80).

## Discussion

In this study, among adults with arthritis, we examined: 1) the association of participant race/ethnicity with meeting PA guidelines and arthritis symptoms; and 2) the association of receipt of a physician recommendation to exercise with meeting PA guidelines and arthritis symptoms, and whether race/ethnicity moderates these associations. Overall, we found that almost 60% of respondents reported at least moderate joint pain and over 40% reported activity limitations, while only 34 and 16% met aerobic and strengthening activity guidelines. Over 40% indicated they had not received a physician recommendation for exercise to relieve symptoms. Controlling for demographic and health characteristics, we found no racial/ethnic differences in the likelihood of meeting aerobic PA guidelines. Controlling for demographic and health factors, African Americans were more likely, and Asians were less likely to meet strengthening guidelines compared to Whites. In adjusted models, Latinos and African Americans were more likely and Asians were less likely than Whites to report severe pain, and there were no differences by race/ethnicity in reporting activity limitations. Receipt of physician recommendation was independently and positively associated with meeting aerobic and strengthening guidelines, having arthritis-associated activity limitations, and more severe joint pain; these associations of receipt of physician recommendation were ubiquitous across all race/ethnic groups except for a weak negative association observed among Latinos for meeting strengthening guidelines.

The proportions of those with arthritis who met aerobic and strengthening guidelines in our study are similar to those found in a previous study of the U.S. population ― approximately 36 and 18%, respectively, supporting the need for more attention to increasing PA in persons with arthritis to relieve symptoms [14]. In our study, disparities among African Americans and Latinos in meeting aerobic PA guidelines observed in bivariate analyses were explained by demographic and health-related factors in the multivariable models. Although in unadjusted analyses, Asians were just as likely as Whites to meet aerobic and strengthening PA guidelines, disparities among Asians in meeting strengthening guidelines emerged after adjusting for demographic and health-related covariates. African Americans were more likely than Whites to meet muscle strengthening guidelines after accounting for demographic and health factors. Unmeasured cultural preferences for weight lifting/strengthening exercises or avoiding over-exertion might help explain these differences among African Americans [24, 25].

In unadjusted analyses, African Americans and Latinos were more likely than Whites to report activity limitations and severe joint pain (and in adjusted analysis). Although African Americans and Latinos were more likely to report arthritis-associated activity limitations in unadjusted models, these differences were attenuated when controlling for demographic and health factors. African Americans and Latinos reported more, and Asians less severe pain than Whites, before and after adjusting for other factors, suggesting that factors other than health and demographic characteristics explain these racial/ethnic differences in levels of pain. Our results agree with prior literature on greater pain among African Americans and Latinos but differ in that we found no racial/ethnic differences on activity limitations [26]. However, our analyses controlled for demographic and health characteristics while the referenced study only included demographics.

Smoking and obesity were negatively associated with meeting aerobic and strengthening guidelines and positively associated with activity limitations and severity of joint pain, in adjusted models. Physician counseling regarding these risk factors is important it has can promote a healthier lifestyle and improved quality of life [27, 28]. Additionally, more severe psychological distress was positively associated with greater arthritis symptom burden (activity limitations and greater pain severity), in adjusted models. Because the relationship between distress and chronic pain is well-established [29] and may be stronger among African Americans and Latinos, [30] screening patients with chronic arthritis-related pain for psychological distress may be indicated.

Our findings are consistent with a 2013 study showing that receipt of physician recommendation to exercise was independently associated with meeting PA guidelines, corroborating the importance of physician advice [16]. Although the proportion of adults with arthritis receiving a physician recommendation to exercise has improved from 28% between 1999 and 2000 [31] to nearly 60%, [16, 32] there is still considerable room for improvement. In our study, in unadjusted analyses, a greater proportion of African Americans, Latinos, and Asians received a physician recommendation to exercise, compared to Whites. In adjusted analyses, both reporting activity limitations and severe pain were associated positively with receipt of an exercise recommendation, which could explain the higher rates of physician recommendation in patients where the need is greatest.

As a retrospective cross-sectional study, this study is limited in that directionality and causality cannot be determined. This study uses secondary data from the NHIS which limits our ability to ask specific questions about cultural preferences and physician-patient interactions that may help explain our findings. Also, the survey item on which the case definition for arthritis was based included gout, lupus, or fibromyalgia in the item stem. Although the prevalence of these conditions is much lower than that of arthritis, we were unable to identify and drop cases of gout, lupus or fibromyalgia from our estimates. Further, our arthritis population had a 14% missing response rate on study outcomes, and it is possible that imputation may have introduced some selection bias.

## Conclusions

Our data suggest that physicians’ exercise recommendations are associated with meeting PA guidelines regardless of race/ethnicity and that both rates of receipt of physician recommendation and adherence to PA guidelines among persons with arthritis need improvement. With the arthritis population expected to increase by over 30 million in 20 years, it is imperative that we reduce disability among this population, especially among Latinos and African Americans [33]. Barriers to exercise must be better characterized among the arthritis community. Finally, although the importance of PA to reduce arthritis symptoms is clear, social, and biological mechanisms that might explain disparities in arthritis-related symptoms need to be better understood.

## Data Availability

The datasets generated during and/or analyzed during the current study are available in the National Health Interview Survey (NHIS) repository, [https://www.cdc.gov/nchs/nhis/data-questionnaires-documentation.htm].

## Abbreviations

PA: Physical Activity
NHIS: National Health Interview Survey
